# The effect of prebiotic and probiotic food consumption on anxiety severity: a nationwide study in Korea

**DOI:** 10.3389/fnut.2024.1385518

**Published:** 2024-05-28

**Authors:** Hyejin Tae, Tae-Suk Kim

**Affiliations:** ^1^Stress Clinic, Health Promotion Center, Seoul St. Mary’s Hospital, The Catholic University of Korea, Seoul, Republic of Korea; ^2^Department of Psychiatry, Seoul St. Mary’s Hospital, The Catholic University of Korea, Seoul, Republic of Korea

**Keywords:** probiotics, prebiotics, microbiome, anxiety, Korea National Health and nutrition examination survey (KNHANES)

## Abstract

**Objectives:**

Over the past decade, research has reported that diet and gut health affect anxiety symptoms through changes in the gut microbiota. Therefore, the introduction of prebiotic and probiotic food favorable for the intestinal microbiota is necessary to improve the mental health of the host. The purpose of this study was to examine the contribution of prebiotic and probiotic foods to lowering anxiety symptoms using a large, nationwide population-based database.

**Materials and methods:**

The study population included 4,317 individuals 19 to 64 years of age who participated in the Korean National Health and Nutrition Examination Survey (KNHANES VII-3, 2019–2021). A food frequency questionnaire was used to evaluate prebiotic and probiotic food consumption. The Generalized Anxiety Disorder Assessment 7-item scale (GAD-7) assessed the severity of anxiety symptoms. The effect of prebiotic and probiotic food consumption on anxiety severity was analyzed using multiple logistic regression.

**Results:**

Anxiety symptom severity was significantly lower in the highest prebiotic and/or probiotic food consumption tertiles compared to the lowest food consumption tertile. We also found a sex difference in the odds ratio for anxiety symptoms. The consumption of prebiotic food was significantly associated with the highest odds of anxiety among both men and women. However, probiotic food had a significant beneficial effect on lowering anxiety symptoms in men but not in women.

**Conclusion:**

Our finding suggests that prebiotic and probiotic food consumption might confer a beneficial influence on anxiety symptoms. Further research is required for a deeper understanding into the mechanisms of the positive effects of prebiotics and probiotics on anxiety.

## 1 Introduction

Anxiety disorder appears to be highly prevalent globally (1). The number of diagnoses have increased due, in part, to greater a perception of disease symptom manifestations but also to the pace of modern life and changes in the population and society (2). Many studies have been conducted about diet and mental health in the past decade. Evidence from previous research has shown that the importance of specific dietary patterns in relation with anxiety. For example, high-fat and high-sugar Western diets are associated with growing risk of mental illness including anxiety and depressive disorders (3, 4), while adherence to the Mediterranean dietary pattern can reduce the risk of several mental illness (5). Other research suggested that sugar, artificial sweeteners, and processed vegetable oils are associated with anxiety, while omega-3 fatty acids, vitamin D, and high protein diets are thought to be potential risk reducing dietary patterns (6). Several systematic studies supported evidence that nutrition is a modifiable factor for intervention in mental illness. (7).

Growing studies have indicated that anxiety symptoms could be regulated by nutrition through gut microbiome alterations and inflammation (8). Specific nutritional strategies affecting the gut microbial ecosystem and regulating inflammation have been reported to either increase or decrease anxiety levels. It has been suggested that the microbiota interactions that influence inflammatory mediators in the immune system, which might lead to chronic inflammation, impairment in the normal functioning of the brain, and anxiety symptoms (9). The focus on the neurobiology of anxiety and central nervous system (CNS) connectivity in the gut has identified promising mechanisms where microbial interference can induce signals that regulate emotions and behaviors through fear and reward anticipation circuits (2).

A mutualistic and symbiotic relation exists between humans and the gut microbiota (10). The gut microbiota has been thought to have interaction with the host central nervous system (CNS) through the microbiota-gut-brain axis (8). The gut microbiota demonstrated extensive effects on brain development, function, and behaviors in animal models (11). Gut microorganisms can be adjusted by dietary measures such as consuming probiotics and prebiotics (12). Probiotics are defined as live microorganisms that promote a healthy gastrointestinal tract and a healthy immune system of the host (13). They are available in foods, including fermented vegetables and fermented dairy products (14). Several studies suggested that probiotics in a healthy diet were promising anxiolytic agents (15). Other preclinical studies demonstrated that probiotic intake increased the level of neurotransmitters including gamma-aminobutyric acid (GABA) (16), serotonin, and its precursor (17), and brain-derived neurotrophic factor (BDNF) (18) in mood disorders. Prebiotics are defined as substrates that foster growth and/or activity of host microorganisms (19), such as non-digestible carbohydrates or plant polyphenols, that provide a health benefit. Foods with notable prebiotic content included fruits, vegetables, and other edible plants, which are good sources of carbohydrates (20). Prebiotics and probiotics have an influence on the relation between the microbiota and the brain and are defined as “psychobiotics” (15). They are regarded as having not only cognitive, emotional, and systemic effects but also anxiolytic effects (21).

To date, research has primarily focused on strategies to introduce potential advantageous microbes in the form of probiotics or through the consumption of prebiotics from food sources. However, evidence supporting that specific probiotics and prebiotics can promote mental health remains limited (22). While nutritional interventions have been considered to improve normal CNS function (23), estimating the impact of food exposure at a large population level is demanding. This is partially owing to the small sample size problems, inhomogeneous probiotic strains, and the diversity in duration of consumption. There are also other challenges in identifying the influence of diet on mental health, especially in controlling the confounding factors of other lifestyles, for example, physical activity, social support networks, and culture. In this respect, we performed a large, nationwide population-based cohort study to identify the regulatory effect of nutritional interventions, especially prebiotics and probiotics, on anxiety. The objective of this study was to examine the effect of prebiotic and probiotic foods consumption on low anxiety severity using a well-controlled, population-based data.

## 2 Methods

### 2.1 Data source and study population

We analyzed large population-based, nationwide data sets from the Korean National Health and Nutrition Examination Survey (KNHANES VII-3), 2019–2021, obtained by the Korea Centers for Disease Control and Prevention. The KNHANES is a surveillance system that evaluates the health condition, dietary habits, and dietary intakes and patterns of a representative nationwide sample. All KNHANES surveys were approved by the Institutional Review Board (IRB) of the Korea Centers for Disease Control and Prevention, and all participants signed a written informed consent prior to study enrollment (IRB: 2018-01-03-5C-A).

The KNHANES consists of three principal components: a health interview, a nutrition survey, and physical examinations (24). The surveys collect a number of variables including demographic characteristics, diet and health-related variables, anthropometric measures, and biochemical profiles. We initially included 7,090 participants who completed the health interview, physical examination, and food frequency questionnaire (FFQ). The study population consisted of individuals aged over 19 years. Those who did not complete the Generalized Anxiety Disorder-7 (GAD-7) assessment, did not have information on the frequency of probiotic and prebiotic food intake, and lacked other interview information were excluded (*n* = 2,773). Finally, 4,317 participants (1852 men and 2,465 women) were included in the analysis.

### 2.2 Assessment of prebiotic and probiotic food consumption

Information on the consumption of prebiotic and probiotic foods was collected by the FFQ. The FFQ have the advantage of being cost effective and convenient in a large-scale epidemiological study. The overall performance of FFQ appears to be an acceptable tool for measuring the nutrient intakes in the Korean population (25). The validity and reproducibility of the FFQ have been described elsewhere (26). The FFQ in the KNHANES included 112 food items to estimate the frequency of consuming. The types of prebiotic food included fruits (excluding jams, sweetened fruits, and juices) and raw vegetables (non-salted or non-starchy vegetables). The types of probiotic food contained fermented vegetables, e.g., fresh pickles, kimchi, sauerkraut, and other kinds of fermented vegetables. We classified prebiotic and probiotic food into two groups: fruits and raw vegetables (prebiotic food, P1) and raw and fermented vegetables (prebiotic and probiotic food, P2). We tried to identify the anxiolytic effect of prebiotic foods (P1), and additive beneficial effects of probiotic foods by comparing prebiotic foods (P1) and prebiotic and probiotic foods (P2). Participants were divided into three groups according to the reported frequency of consuming prebiotic and probiotic foods (Q1 the lowest tertile, Q2 the middle tertile, Q3 the highest tertile).

### 2.3 Estimation of anxiety

The GAD-7 assessment is a practical, 7-item self-reported questionnaire frequently used to measure the severity of anxiety symptoms in primary care and research settings (27). The original validation of the GAD-7 in a large clinical population showed that the measure has good reliability (Cronbach’s alpha = 0.92; test–retest-reliability intraclass correlation = 0.83), and good levels of criterion and factorial validity. Total scores range from 8 to 40, with higher scores suggesting higher levels of anxiety. A recommended cut-off point indicating a high likelihood of GAD is 10 or greater, and scores of 5, 10, and 15 are suggested to represent mild, moderate, and severe levels of anxiety. A score of 10 or greater on the GAD-7 represents a reasonable cut-off value for optimizing sensitivity (89%) and specificity (82%) compared to a mental health professionals diagnosis using the Diagnostic and Statistical Manual of Mental Disorders (DSM)-V diagnostic criteria and a structured psychiatric interview. The Korean version of GAD-7 has been considered as a reliable and valid instrument in measuring anxiety (28).

### 2.4 Statistical analyses

The KNHANES was performed using a representative nationwide sample of the Koreans applying a complex multistage stratified sampling method. This study utilized stratification, clustering, and sample weight variables for the statistical analysis and data management using SPSS version 21.0 for Windows (SPSS, Inc., and IBM Company, Chicago, IL, United States).

The participants were classified into either the high anxiety group (HA) or the low anxiety group (LA) based on the cut off of total GAD-7 scores. The demographic and clinical characteristics of the individuals were shown as means (standard deviation [SD]) for continuous variables and as numbers (%) for categorical variables. Two-tailed tests were used in all cases, and statistical significance was defined as *p* < 0.05, with confidence intervals at 95%. Relationships between each of these variables and anxiety were demonstrated by an independent samples t-test for continuous variables and a chi-squared test for categorical variables.

Based on prior studies (29, 30), we identified potential confounders, including age, sex, marital status, educational level, household income, lifestyle (smoking status, drinking status, aerobic exercise, and anaerobic exercise), daily energy intake, and body mass index (BMI). Participants were also classified by educational level as follows: less than elementary school, middle school, high school, and college or more. Participants were divided into monthly household income quartiles as follows: the lowest (Q1), lower middle (Q2), upper middle (Q3), and the highest (Q4). Smokers were defined as persons who were currently smoking and had ever smoked >100 cigarettes in their entire life. Otherwise, the subjects were considered as non-smokers. Drinkers were defined as those who at least once a month in the past 12 months. Aerobic exercise was defined by responses to the question, “How many days and time do you spend in physical activity such as walking?” “How many days and time do you spend in strength training, such as push-ups, sit-ups, or lifting barbells or dumbbells, to develop muscles?” was an example of a question about anaerobic exercise. Individuals who reported participating in physical activity at least 5 times a week and more than 30 min each time were regarded as active. On the other hand, those who exercised but at a level of physical activity that failed to meet the criteria were regarded as inactive. The FFQ provided detailed information on BMI and daily total energy.

We conducted multivariate logistic regression analysis to investigate the effect of prebiotic and probiotic food consumption (the lowest, middle, and highest) on anxiety severity (high/low). The association between fruit and raw vegetable (prebiotic food, P1) consumption and anxiety and raw and fermented vegetable (prebiotic and probiotic food, P2) consumption and anxiety were determined by logistic regression analysis. This model is widely applied in epidemiological studies to examine the relationship between independent variables and binary outcomes. Three models were applied: Model 1 (unadjusted), Model 2 (adjusted for age and sex), and Model 3 (fully adjusted for were age, sex, body mass index, marital status, educational level, household income level, smoking status, drinking status, aerobic exercise, anaerobic exercise, and mean daily energy intake). We calculated odds ratios (ODs; Colica et al.) and 95% confidence intervals (CIs; Sánchez-Villegas et al.) using the lowest prebiotic and/or probiotic food consumption tertile groups as references. A backward stepwise selection was employed to select significant covariates. The Hosmer-Lemeshow goodness-of-fit test was performed to assess the goodness of fit of the logistic regression. Differences were considered statistically significant for *p* values under 5% (*p* < 0.05) in two-sided tests. We also performed a subgroup analysis by gender.

## 3 Results

### 3.1 General characteristics

The general characteristics of the individuals according to anxiety severity assessed by the GAD-7 are presented in Table 1. The LA and HA groups composed 85.0% (n, 3,671) and 15.0% (n, 646), respectively, of the total. The LA participants had a mean age of 54.5 (±16.9) years, with 2033 females (55.4%) and 1,638 males (44.6%). The HA participants were significantly younger, with an average age of 49.2 (±17.6) years, and included 432 females (66.9%) and 214 males (33.1%). The sex ratio and marital status were significantly different in the two groups.

**Table 1:** Characteristics of subjects according to the severity of anxiety.

Characteristic	Low anxiety (LA)	High anxiety (HA)	*p*-value*^a^*
Participants, N (%)	3,671 (85.0)	646 (15.0)	
Age (years), mean ± SD	54.5 ± 16.9	49.2 ± 17.6	0.00^**^
Sex			
Male, N (%)	1,638 (44.6)	214 (33.1)	0.00^**^
Female, N (%)	2,033 (55.4)	432 (66.9)	
Marital status			
Married, N (%)	3,041 (82.8)	470 (72.8)	0.00^**^
Single, N (%)	630 (17.2)	176 (27.2)	
Educational level			
Elementary or less, N (%)	719 (19.6)	118 (18.3)	0.58
Middle school, N (%)	385 (10.5)	60 (9.3)	
High school, N (%)	1,202 (32.7)	214 (33.1)	
College or more, N (%)	1,365 (37.2)	254 (39.3)	
Household income			
Q1 (lowest), N (%)	727 (19.8)	133 (20.6)	0.78
Q2, N (%)	875 (23.8)	163 (25.2)	
Q3, N (%)	1,003 (27.3)	169 (26.2)	
Q4 (highest), N (%)	1,066 (29.0)	181 (28.0)	
Smoking status			
No, N (%)	3,133 (85.3)	533 (82.5)	0.06
Yes, N (%)	538 (14.7)	113 (17.5)	
Drinking status			
No, N (%)	1,257 (34.2)	208 (32.2)	0.31
Yes, N (%)	2,414 (65.8)	438 (67.8)	
Aerobic exercise			
No, N (%)	600 (16.3)	111 (17.2)	0.60
Yes, N (%)	3,071 (83.7)	535 (82.8)	
Anaerobic exercise			
No, N (%)	2,685 (73.1)	497 (76.9)	0.04*
Yes, N (%)	986 (26.9)	149 (23.1)	
Daily energy intake (kcal/d), mean ± SD	1,801.0 ± 754.4	1,708.7 ± 783.5	0.00^**^
BMI (kg/m^2^), mean ± SD	24.1 ± 3.7	23.8 ± 3.9	0.04*
Fruits*^b^*			
Q1 (lowest), N (%)	465 (12.7)	110 (17.0)	0.00^**^
Q2, N (%)	1,724 (47.0)	331 (51.2)	
Q3 (highest), N (%)	1,482 (40.4)	205 (31.7)	
Raw vegetables			
Q1 (lowest), N (%)	530 (14.4)	148 (22.9)	0.00^**^
Q2, N (%)	1,385 (37.7)	260 (40.2)	
Q3 (highest), N (%)	1,756 (47.8)	238 (36.8)	
Raw & fermented vegetables*^c^*			
Q1 (lowest), N (%)	326 (8.9)	100 (15.5)	0.00^**^
Q2, N (%)	1,415 (38.5)	278 (43.0)	
Q3 (highest), N (%)	1,930 (52.6)	268 (41.5)	

N, number of subjects; SD, standard deviation; Household income levels were divided into quartiles: Q1, below the 25th percentile; Q2, 26th to 50th percentile; Q3, 51th to 75th percentile; and Q4, 76th to 100th percentile; Frequency of consuming fruits, raw vegetables, and raw & fermented vegetables was divided into tertiles: Q1, the lowest tertile; Q2, the middle tertile; and Q3, the highest tertile. ^a^Statistical significance from independent t-tests or Chi-square tests. ^b^Excluding preserved in sugar such as jam and fruit juice. ^c^Including fresh pickles, Kimchi, Sauerkraut and other kinds of fermented vegetables. *p < 0.05, **p < 0.01.

The HA groups reported significantly different characteristics with lower anaerobic exercise (*p* = 0.04), lower daily energy intake (*p* = 0.00), and lower BMI (*p* = 0.04). However, no significant differences in educational level, household income, alcohol and nicotine use, and aerobic exercise were shown between two groups. We classified participants into tertile groups according to the frequency of prebiotic and probiotic food intake. The LA groups showed significantly different intake patterns, consuming more fruits (*p* = 0.00), rawer vegetables (*p* = 0.00), and rawer and fermented vegetables (*p* = 0.00). Additionally, frequency distribution of consuming fruits, raw vegetables, and raw & fermented vegetables according to the severity of anxiety is displayed in Supplementary Figure S1.

### 3.2 Relationship between prebiotic and/or probiotic food consumption and anxiety

We examined the relationship between prebiotic and/or probiotic food consumption and anxiety symptoms (Table 2). We performed logistic regression analysis for each prebiotic and probiotic food consumption group and anxiety severity according to GAD-7 scores. First, we examined the association between prebiotic food (P1) and anxiety severity. Subjects in the highest tertile of prebiotic food consumption reported significantly lower severity of anxiety symptoms than those in the middle and lowest tertiles (unadjusted OR = 0.52; 95% CI: 0.41–0.65; Table 2). In Model 2, adjusted for age and sex, the association between prebiotic food consumption and anxiety remained significant statistically (adjusted OR = 0.60; 95% CI: 0.47–0.77), with a fully adjusted OR of 0.66 (95% CI: 0.51–0.85). These results demonstrated a decreasing odds ratio for anxiety with increases in prebiotic food intake (*p* for trend <0.05). Second, regarding prebiotic and probiotic food (P2) consumption, the highest food consumption tertile group had significantly lower anxiety severity in the unadjusted (OR = 0.45; 95% CI: 0.35–0.59) and fully adjusted logistic regression models (OR = 0.67; 95% CI: 0.50–0.89). These findings suggested that the odds ratio of anxiety decreased significantly as the intake of prebiotic and probiotic foods increased.

**Table 2:** Multiple logistic regression analysis for the association between prebiotic and/or probiotic food consumption and anxiety symptoms.

Anxiety symptoms	Prebiotic food consumption (P1)		
	Q1	Q2	Q3
Model 1^a^	Reference	0.62 (0.51–0.75)**	0.52 (0.41–0.65)**
Model 2^b^	Reference	0.69 (0.56–0.84)**	0.60 (0.47–0.77)**
Model 3^c^	Reference	0.73 (0.59–0.90)**	0.66 (0.51–0.85)**
	Prebiotic & probiotic food consumption (P2)		
	Q1	Q2	Q3
Model 1	Reference	0.64 (0.50–0.83)**	0.45 (0.35–0.59)**
Model 2	Reference	0.71 (0.55–0.92)*	0.62 (0.47–0.82)**
Model 3	Reference	0.76 (0.58–0.99)*	0.67 (0.50–0.89)**

A total of 4,317 participants with complete data were included in the analysis; p value 0.954 for the Hosmer-Lemeshow goodness-of-fit test, which did not indicate significant poor fit; Anxiety symptoms were estimated by the Generalized Anxiety Disorder-7 (GAD-7) score; Q1, Q2 and Q3 correspond to the lowest, middle and highest tertile assigned by frequency of consuming prebiotic and probiotic foods; Values are odds ratio (95% confidence interval). ^a^Model 1: Crude. ^b^Model 2: Adjusted for age and sex. ^c^Model 3: Adjusted for age, sex, body mass index, marital status, educational level, household income level, smoking status, drinking status, aerobic physical activity, anaerobic physical activity, and daily energy intake. *p < 0.05, **p < 0.01.

### 3.3 Subgroup analysis by gender

We conducted a subgroup analysis of men and women to examine sex differences in the association between prebiotic and probiotic food consumption and anxiety symptoms. There was a remarkable association between each type of food consumption and anxiety in men, with a significantly lower OR in all models (Supplementary Table S1). After fully adjusting for the confounders in model 3, the OR for prebiotic food consumption was 0.60 (95% CI: 0.37–0.97) and prebiotic and probiotic food consumption was 0.53 (95% CI: 0.33–0.86; Table 3). The consumption of both prebiotic and probiotic food (raw and fermented vegetables, P2) was significantly related to the highest odds of anxiety among men compared to consuming only prebiotic food (fruit and raw vegetables, P1). In contrast, no significant relationship was found in women for prebiotic and probiotic food (P2) consumption (Table S2). Anxiety symptoms in women were only significantly lower in the highest prebiotic food (P1) consumption tertile in the fully adjusted model (adjusted OR = 0.66; 95% CI: 0.49–0.90; Table 3).

**Table 3:** Multiple logistic regression analysis for the association between prebiotic and/or probiotic food consumption and anxiety symptoms by sex (in the Model 3).

Anxiety symptoms	Prebiotic food consumption (P1)		
	Q1	Q2	Q3
Men (*n* = 1852)	Reference	0.61 (0.42–0.87)**	0.60 (0.37–0.97)*
Women (*n* = 2,465)	Reference	0.78 (0.61–1.00)	0.66 (0.49–0.90)**
Group	Prebiotic & probiotic food consumption (P2)		
	Q1	Q2	Q3
Men	Reference	0.55 (0.36–0.85)**	0.53 (0.33–0.86)**
Women	Reference	0.86 (0.62–1.20)	0.72 (0.50–1.02)

A total of 1,852 men and a total of 2,465 women with complete data were included in the analysis; p value 0.299 (men) and p value 0.247 (women) for the Hosmer-Lemeshow goodness-of-fit test, which did not indicate significant poor fit; Anxiety symptoms were estimated by the Generalized Anxiety Disorder-7 (GAD-7) score; Q1, Q2 and Q3 correspond to the lowest, middle and highest tertile assigned by frequency of consuming prebiotic and probiotic foods; Values are odds ratio (95% confidence interval). Model 3: Adjusted for age, sex, body mass index, marital status, educational level, household income level, smoking status, drinking status, aerobic physical activity, anaerobic physical activity, and daily energy intake. *p < 0.05, **p < 0.01.

## 4 Discussion

This study used 2019–2021 KNHANES data to explore the relationship between prebiotic and/or probiotic food consumption and anxiety symptoms in a representative national sample of the South Korean population. Individuals with high anxiety symptoms showed significant difference in lifestyle patterns, with higher nicotine use, lower anaerobic activity, lower daily energy intake, and lower BMI. Although we could not exclude the possibility that people with healthier lifestyle patterns are inclined to change their behaviors to improve mental health, prebiotic and probiotic effects remained significant in lowering the risk for anxiety after adjusting for socio-demographic characteristics. In the present study, higher prebiotic and/or probiotic food consumption was significantly associated with lower anxiety symptoms severity reported on the GAD-7 questionnaire.

The following mechanisms have been proposed the positive effects of prebiotics on anxiety. Prebiotics may relieve anxiety symptoms by encouraging the proliferation of beneficial microbes and discouraging the growth of pathogenic microbes (31). The prebiotics commonly studied for reducing anxiety include fructooligosaccharides and galactooligosaccharides, which may be related to the modulation of cortical IL-1b and 5-HT2A (5-hydroxytryptamine2A) receptor expression (32). In addition, both prebiotics may have anxiolytic effects by enhancing *Bifidobacterium* in humans (33). Bifidobacteria can produce mainly lactate and acetate, which can be changed to short-chain fatty acids (SCFAs) by other bacterial species (34). The SCFAs butyrate, propionate, and acetate are important products of bacterial fermentation in the human intestine and are known to have the ability to regulate anxiety-like behavior (35). Emerging research has demonstrated that SCFAs may regulate the effects of gut bacteria on stress responses of the HPA axis and attenuate the cortisol response to acute psychosocial stress (36).

Probiotics have been suggested to have the potential to change brain function through several mechanisms. Dietary interventions with probiotics may increase diversity of intestinal microbiome and improve mental health outcomes. Probiotics can modulate neurotransmitters and proteins (GABA, glutamate, histamine, serotonin, and BDNF) by gut-brain axis, and are essential to control the neural excitatory-inhibitory balance, mood, and anxiety (37, 38). Bravo et al. (16) identified that ingestion of the lactic acid bacteria *Lactobacillus rhamnosus* resulted in vagus nerve dependent anxiolytic behavioral effects and change of GABA receptor expression. Another potential mechanism of action of probiotics is their anti-inflammatory properties (39). Probiotics may reduce inflammatory cytokines and oxidative stress markers (40), and up-regulate plasma IL-10, which has anti-inflammatory effects (41). It has been reported that oxidative imbalance and inflammation have an important role in the pathogenesis of anxiety disorder (42).

There are only a few studies investigating the relationship between consumption of fermented foods and anxiety. Sousa et al. (43) demonstrated that the consumption of fermented dairy products such as yogurt and cheese has a positive effect on reducing anxiety in Portuguese students. A prospective cohort study indicated that a positive association was found between the consumption of fermented food such as yogurt, kefir, and soured milk and severity of anxiety symptoms in Polish adults (44). In comparison, Koreans intake a relatively high amount of fermented vegetables. Above all, Kimchi is a contributory factor in 40–45% of the daily total vegetable consumption of Koreans (45). Kimchi, a traditional Korean fermented vegetable product, is made from mixing and fermenting various vegetables and known for potential diverse lactic acid bacteria (LAB) sources (46). Among them, *Lactobacillus plantarum* has been suggested to be responsible for the late stage of kimchi fermentation through the production of organics (47). Paying attention to the antibacterial and immune regulatory characteristics of *L. plantarum*, some clinical studies have examined its probiotic properties (48) and reported the alleviation of stress and anxiety (49). In this study, we also demonstrated that fermented probiotic food, including kimchi, had a significant beneficial effect in lowering anxiety symptoms.

We also found a sex difference in OR for anxiety symptoms. We demonstrated that fermented probiotic food had a significant beneficial effect on lowering anxiety symptoms in men but not in women. Sex-related differences are well-established in anxiety disorders. Although psychological and cultural factors can make the sex differences, biological factors are also identified to play an important role (50). Biological factors might have contributed to the sex-related differences in this study. Sex gap might be attributed to the genetic predisposition, differences in brain circuitry (50), and fluctuating levels of gonadal steroids in women across the menstrual cycle (51). In particular, sex hormones may play a pivotal role in the microbiota composition, which may increase pro-inflammatory mediators and adverse psychological responses (52). For example, women are reported to have a lower *Bacteroides abundance* in their gut microbiome compared to men (53). Significant decreases in the abundance of *Bacteroides* have been showed in the fecal microbiota of individuals with psychological distress (54). Additionally, previous studies have indicated that male and female gut microbiota respond differently to dietary manipulation, and the male microbiota might be more affected by dietary intake than females (55). Sex disparities in the relationship between dietary intake and anxiety status could be attributed to the sex differences in microbial composition (56), but further studies are needed to improve our understanding of how the mechanism of gut microbiota may relate to gender differences in anxiety symptoms.

The interpretation of the present study results should consider the following limitations. First, we used self-reported measures to estimate dietary intake and anxiety symptoms. Self-reporting can encourage participants to overstate severity of symptoms and amount of intake. Second, the estimated food intake might not fully represent participants’ usual food consumption because this survey utilized 3-day dietary records. Third, we cannot confirm causal relationships between prebiotic and probiotic food intake and anxiety because of the cross-sectional design of the study. Because the exposure and outcome are simultaneously assessed, it is difficult to draw predictive conclusions based on these differences. A longitudinal cohort study is guaranteed to confirm the causal relationship suggested by our findings. Fourth, we did not identify how variables interact or which variables interact. This is a limitation that points to the need for the next step in improving our model. Fifth, we could not consider the use of probiotic and prebiotic supplements. When taking into account that supplement usage has become increasingly popular, evaluating the impact of probiotic and prebiotic supplements related to anxiety deserves further study. In addition, we could not analyze data on using only probiotic food because the KNHANES did not contain sufficient information. Therefore, further research might help us evaluate various aspects of probiotic food using different scales and in-depth clinical interviews. Sixth, we could not estimate the microorganism’s dose–response relations because of the lack of relevant information on the bacterial content of foods.

In conclusion, our finding suggests that prebiotic and probiotic food consumption is associated with low anxiety severity. Probiotic organisms are essential to maintain an exquisite balance in intestinal microbiota. Many previous studies confirmed probiotics could improve host health. Prebiotics may be utilized as a promising alternative to probiotics supporting the beneficial effects of them. Thus, proper selection of probiotic strains and prebiotics supplementation may contribute to improving overall effect of probiotics in the gastrointestinal tract, which may confer a beneficial influence on mental health. Further multidisciplinary research is needed to obtain a deeper understanding of the biological mechanisms supporting the observed relationship between prebiotics, probiotics and anxiety.

## Data availability statement

Publicly available datasets were analyzed in this study. This data can be found at: https://knhanes.kdca.go.kr/knhanes/eng/index.do Korean National Health and Nutrition Examination Survey (KNHANES).

## Author contributions

HT: Conceptualization, Data curation, Formal analysis, Investigation, Methodology, Software, Validation, Visualization, Writing – original draft, Writing – review & editing. T-SK: Conceptualization, Supervision, Validation, Writing – review & editing.
