# Supplementary material for: The effect of prebiotic and probiotic food consumption on anxiety severity: a nationwide study in Korea
Source: Front Nutr. 2024 May 28;11:1385518. doi: 10.3389/fnut.2024.1385518 (PMC11165345; doi:10.3389/fnut.2024.1385518)
Supplement: Supplementary file 1 [file Data_Sheet_1.pdf]

Supplementary Tables:

Table S1. Multiple logistic regression analysis for the association between prebiotic and/or probiotic food consumption and anxiety symptoms in men.

Anxiety symptoms	Prebiotic food consumption (P1)		
	Q1	Q2	Q3
Model 1	Reference	0.50 (0.35-0.70)**	0.43 (0.27-0.67)**
Model 2	Reference	0.59 (0.41-0.85)**	0.57 (0.36-0.92)*
Model 3	Reference	0.61 (0.42-0.87)**	0.60 (0.37-0.97)*
	Prebiotic & probiotic food consumption (P2)		
	Q1	Q2	Q3
Model 1	Reference	0.50 (0.32-0.76)**	0.35 (0.23-0.54)**
Model 2	Reference	0.55 (0.36-0.86)**	0.52 (0.32-0.83)**
Model 3	Reference	0.55 (0.36-0.85)**	0.53 (0.33-0.86)**

Note: A total of 1,852 men with complete data were included in the analysis; *p* value 0.299 for the Hosmer-Lemeshow goodness-of-fit test, which did not indicate significant poor fit; Anxiety symptoms were estimated by the Generalized Anxiety Disorder-7 (GAD-7) score; Q1, Q2 and Q3 correspond to the lowest, middle and highest tertile assigned by frequency of consuming prebiotic and probiotic foods; Values are odds ratio (95% confidence interval).

Model 1: Crude

Model 2: Adjusted for age and sex

Model 3: Adjusted for age, sex, body mass index, marital status, educational level, household income level, smoking status, drinking status, aerobic physical activity, anaerobic physical activity, and daily energy intake

*$p < .05$, **$p < .01$.

Table S2. Multiple logistic regression analysis for the association between prebiotic and/or probiotic food consumption and anxiety symptoms in women.

Anxiety symptoms	Prebiotic food consumption (P1)		
	Q1	Q2	Q3
Model 1	Reference	0.67 (0.53-0.85)**	0.52 (0.39-0.69)**
Model 2	Reference	0.75 (0.58-0.96)*	0.62 (0.46-0.83)**
Model 3	Reference	0.78 (0.61-1.00)	0.66 (0.49-0.90)**
	Prebiotic & probiotic food consumption (P2)		
	Q1	Q2	Q3
Model 1	Reference	0.75 (0.54-1.03)	0.53 (0.39-0.74)**
Model 2	Reference	0.82 (0.59-1.14)	0.68 (0.48-0.97)*
Model 3	Reference	0.86 (0.62-1.20)	0.72 (0.50-1.02)

Note: A total of 2,465 women with complete data were included in the analysis; *p* value 0.247 for the Hosmer-Lemeshow goodness-of-fit test, which did not indicate significant poor fit; Anxiety symptoms were estimated by the Generalized Anxiety Disorder-7 (GAD-7) score; Q1, Q2 and Q3 correspond to the lowest, middle and highest tertile assigned by frequency of consuming prebiotic and probiotic foods; Values are odds ratio (95% confidence interval).

Model 1: Crude

Model 2: Adjusted for age and sex

Model 3: Adjusted for age, sex, body mass index, marital status, educational level, household income level, smoking status, drinking status, aerobic physical activity, anaerobic physical activity, and daily energy intake

*$p < .05$, **$p < .01$.

Supplementary Figures:

Figure S1. Frequency distribution of consuming fruits, raw vegetables, and raw & fermented vegetables according to the severity of anxiety. a. Frequency distribution for the association between consuming fruits and the severity of anxiety. b. Frequency distribution for the association between consuming raw vegetables and the severity of anxiety. c. Frequency distribution for the association between consuming raw & fermented vegetables and the severity of anxiety.

a

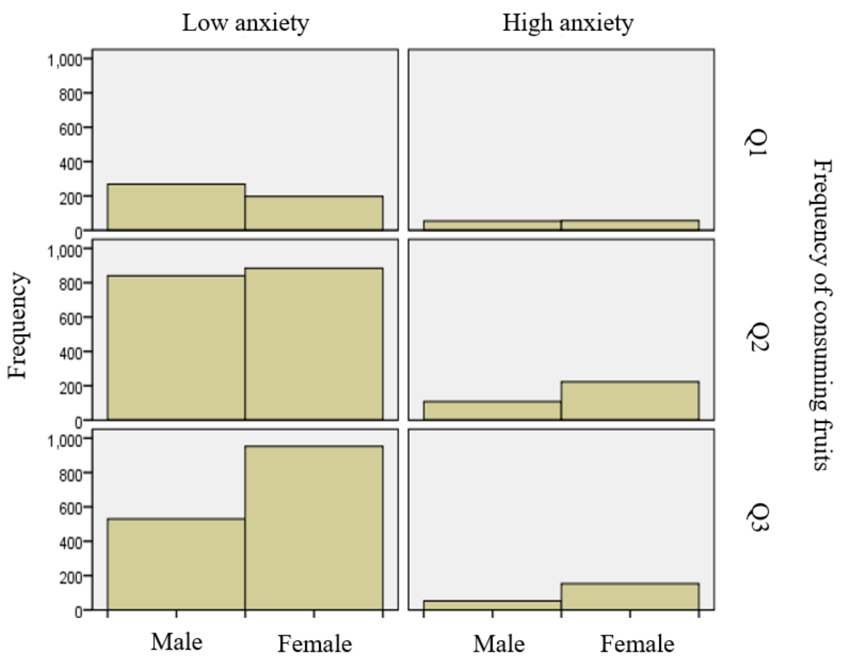

b

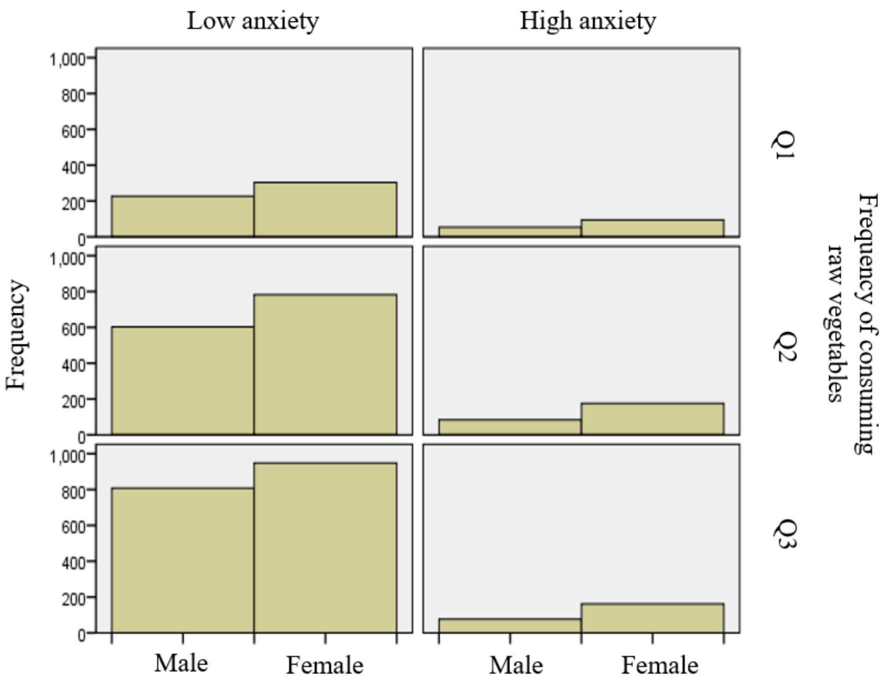

c

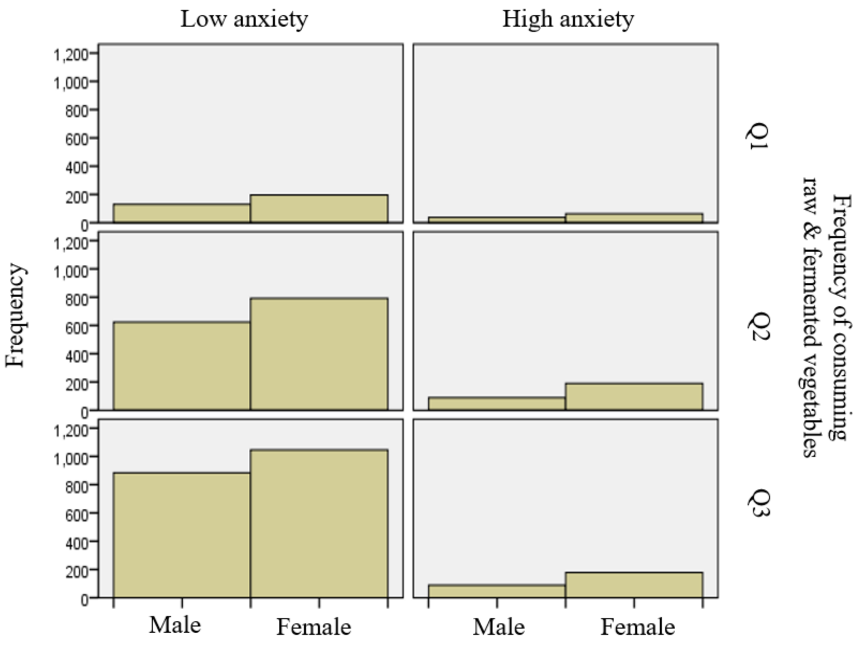
Low anxiety
High anxiety
Frequency
1,200
1,000
800
600
400
200
0
Q1
Q2
Q3
Frequency of consuming raw & fermented vegetables
Male
Female
Male
Female
